# Oral health and salivary inflammatory markers in children and adolescents with type 1 diabetes: A cross-sectional study

**DOI:** 10.1007/s40618-026-02808-4

**Published:** 2026-01-08

**Authors:** Eulalia Catamo, Gianluca Tornese, Chiara Navarra, Luana Aldegheri, Nunzia Zanotta, Manola Comar, Milena Cadenaro, Antonietta Robino

**Affiliations:** 1 Institute for Maternal and Child Health – IRCCS Burlo Garofolo, Via Dell Istria 65, 34137 Trieste, Italy; 2 Department of Medicine, Surgery and Health Sciences, University of Trieste, Piazzale Europa 1, 34127 Trieste, Italy

**Keywords:** Caries, Gingivitis, Inflammatory markers, Oral health, Type 1 diabetes

## Abstract

**Purpose:**

To evaluate the oral health status of children and young adults with type 1 diabetes (T1D) compared with healthy controls (HC), and its association with clinical parameters and salivary inflammatory markers.

**Methods:**

Seventy-two subjects with T1D and 86 HCs underwent a clinical oral examination. Salivary cytokines were measured using multiplex immunoassays. Differences between oral conditions, clinical parameters and cytokines were tested and mediation models were used to evaluate the role of T1D and HbA1c.

**Results:**

T1D subjects showed significantly higher frequencies of caries (67% *vs.* 28.5%) and gingivitis (76% *vs.* 42%) compared with HC (p < 0.001), independently by oral hygiene habits. In T1D, the presence of caries and gingivitis is associated with unfavourable clinical outcomes, including higher HbA1c, triglycerides, and urinary creatinine (p-value < 0.05). Salivary cytokine profile differed according to oral condition: caries was associated with reduced IL-4, IL-10, and IFN-γ; gingivitis with elevated IL-1Ra and RANTES; and both conditions with increased IL-1β (p-value < 0.05). Mediation analyses revealed no significant influence of HbA1c.

**Conclusion:**

Children and young adults with T1D show a higher prevalence of oral diseases and distinct inflammatory profile, regardless of metabolic status. These findings call for routine integration of oral assessment in T1D management.

**Supplementary Information:**

The online version contains supplementary material available at 10.1007/s40618-026-02808-4.

## Introduction

Type 1 diabetes mellitus (T1D) is a chronic autoimmune disease characterized by the destruction of pancreatic β-cells, leading to lifelong insulin dependence and persistent hyperglycaemia, which causes numerous micro and macrovascular complications [1]. T1D subjects also appear to be at increased risk for various oral diseases, including dental caries, gingivitis, and periodontitis [2, 3]. Several mechanisms have been proposed to explain this association, such as impaired neutrophil function, altered cytokine profiles, reduced salivary flow, and elevated levels of advanced glycation end-products (AGEs), all of which may promote oral inflammation and tissue damage [4, 5].

However, evidence regarding the relationship between T1D and oral health remains inconsistent. Some studies have reported higher rates of caries or plaque accumulation in children with T1D [6, 7], whereas others have found no significant associations [8] or have even reported a lower prevalence of caries in this population [9]. Similarly, findings on the relationship between glycaemic control and caries risk are conflicting. While some studies report no association between poor glycaemic control and caries [10, 11], others suggest that optimal glycaemic control is associated with a reduced prevalence of caries [12, 13], supporting the hypothesis that improved glycaemic control may contribute to better oral health outcomes [14].

In contrast, the association between T1D and gingivitis or periodontitis is better documented. A significantly higher incidence of gingivitis in children with T1D compared with the healthy population has been reported, with this difference becoming more evident during adolescence [15]. Similarly, studies found that periodontitis, considered as the sixth chronic complication of diabetes [16], tends to develop earlier in individuals with T1D than in healthy controls, especially during childhood [17] and adolescence [18].

Although the exact mechanisms underlying periodontitis in the context of diabetes are not fully understood, it is hypothesized that several diabetes-related processes, such as microvascular damage, immune dysfunction, increased susceptibility to infections, and alterations in the oral microbiome, may contribute to periodontal inflammation [19, 20]. In turn, periodontitis can impair glycaemic control [21], and hyperglycaemia can further stimulate the production of proinflammatory cytokines, including IL-1β, IL-6, and TNF-α, creating positive feedback that accelerates periodontal bone resorption [22]. This evidence supports the presence of a bidirectional relationship between periodontal inflammation and glycaemic control [23].

Despite the clinical relevance of oral diseases in young individuals with T1D and the recommendation for regular dental review as part of preventive care [24], most existing studies have focused on adult populations, and data on children and adolescents remain limited. Furthermore, few studies have concurrently examined multiple oral pathologies alongside their associated inflammatory profiles in this age group. Therefore, in the present study, the oral health status of children and young adults with T1D was evaluated in comparison with healthy controls (HC). Additionally, among T1D subjects, the associations of oral diseases with clinical parameters and salivary inflammatory markers were investigated.

## Subjects, materials and methods

### Subjects

A cross-sectional study of 72 T1D and 86 HC subjects was conducted at the Diabetes and Dental Units of the Institute for maternal and child health IRCCS “Burlo Garofolo” (Trieste, Italy) between May 2022 and December 2023 during routine outpatient visits.

Inclusion criteria for T1D participants were: diagnosis for at least 1 year, age between 6 and 21 years, and absence of other types of diabetes mellitus (i.e., type 2, monogenic diabetes, cystic fibrosis-related diabetes). For HC selection, subjects similar to T1D subjects in terms of sex and age were included. Then, subjects with HbA1c > 6% (> 42 mmol/mol), measured with finger pricks using portable instrumentation (QuikRead go, A. De Mori S.p.A., Milan, Italy), were excluded. Furthermore, subjects diagnosed with any other form of diabetes, obesity and other metabolic and autoimmune disorders, and family history of diabetes, were excluded. 

Additionally, all subjects (both T1D and HC) with immunodeficiencies and neoplasms and who had taken antibiotics or anti-inflammatory drugs within 14 days prior to enrolment were excluded.

Each subject who met the eligibility criteria and presented to the clinic during the study period was invited to participate in the study.

At the time of enrolment, demographic data, including age, sex, and anthropometric measurements were collected for all participants. BMI standard deviation scores (BMI-SDS) were calculated using WHO reference charts [25] via the Growth Calculator 4 software (http://www.weboriented.it/gh4/). 

For T1D subjects, clinical data at onset, including age at onset and the presence of ketoacidosis (DKA) (calculated as a venous pH of < 7.3 and/or a bicarbonate (HCO3) level of < 18 mmol/L) [26], type of diabetes therapy, insulin requirements and HbA1c from capillary blood measurements over the past year, were collected by medical records. HbA1c and insulin requirements at recruitment were used to calculate IDAA1c (Insulin-Dose Adjusted A1c) using the formula: HbA1c (%) + 4 × insulin dose (units/kilogram/day) [27]. T1D subjects were categorized into two groups: poor glycaemic control (PGC) and good glycaemic control (GGC) using as cut-off the median HbA1c value over the past year (HbA1c ≥ 7% or < 7%, ≥ or < 53 mmol/mol, respectively) [28].

Finally, data from the most recent visit were retrieved, including: fasting lipid profile (total cholesterol [TC]; low-density lipoprotein cholesterol [LDL-C]; high-density lipoprotein cholesterol [HDL-C]; triglycerides [TG]), serum and urine creatinine, albumin-to-creatinine ratio (ACR). Estimated glomerular filtration rate (eGFR) were calculated using the Schwartz equation: 0.413 × height (cm)/serum creatinine mg/dL [29].

The research project was approved by ethic committee (CEUR-2018-Em-323-Burlo). Before the enrolment all participants or their parents/guardians (for participants aged < 18 years) provided written informed consent.

### Oral-dental assessment

At the time of enrolment, each participant completed a questionnaire on oral hygiene habits, which included questions about the number of daily brushings, the methods used for oral cleaning (manual toothbrush, electric toothbrush, dental floss, etc.), the frequency of dental visits, and the reasons for attending such visits.

The oral clinical examination was conducted using a mirror and a CP12 Periodontal Probe. The oral-dental assessment included the definition of the state of the dentition and dental formula, the state of caries and periodontal health, and salivary pH. Validated standard assessment tools were used. Dental health was assessed by recording the percentage of caries in the dentition (permanent, mixed, deciduous), excluding third molars (range 0–28) out of the total number of teeth in the arch. Unerupted, congenitally lost, or supernumerary teeth, teeth extracted for orthodontic reasons, teeth filled for reasons other than caries, and non-exfoliated deciduous teeth in the permanent dentition were not taken into consideration. Where clinically indicated, intraoral x-rays were performed, or recently performed panoramic x-rays were requested.

Periodontal health was assessed by calculating the bleeding on probing index (BoP), recording, for each tooth, the bleeding sites after gingival probing at six sites (mesial, mid, and distal on both the buccal and lingual surfaces). This number was divided by the total number of sites available in the mouth and multiplied by 100. The BoP index is expressed as a percentage.

The O’Leary Plaque Index (PI) was used to assess the oral hygiene status of individuals. After applying a plaque disclosing agent (Mira-2-Ton®, Hager & Werken GmbH & Co., Duisburg, Germany), the number of stained surfaces was recorded for each tooth. Four surfaces were evaluated for each tooth (distal, mesial, buccal, and lingual). PI was calculated by dividing the total number of surfaces by the surfaces with plaque. Third molars were excluded from this count.

Moreover, BoP (%), PI (%), and Periodontal Pocket Depth (PPD, measured in mm) parameters were assessed, and subjects were classified into three categories: Health (BoP ≤ 10%, PI ≤ 25%, PPD ≤ 3 mm); Plaque-induced gingivitis (BoP > 10%, PI > 25%, PPD ≤ 3 mm); Non-plaque-induced gingivitis (BoP > 10%, PI < 25%, PPD ≤ 3 mm) (Adapted from Chapple ILC, et al., 2018) [30]. Given the extremely small number of subjects with non-plaque-induced gingivitis, these individuals were excluded from the study.

### Cytokines analysis

For each participant 2 mL of saliva were collected and immediately stored at -80 °C until further processing. A panel of cytokines, chemokines and growth factors was assessed in all the saliva samples using magnetic bead-based multiplex immunoassays (Bio-Plex Pro™ human cytokine; Bio-Rad Laboratories, Milan, Italy). Samples were centrifuged al 10,000 g for 10 min at room temperature (RT) prior to analysis. Assays were performed according to the manufacturer’s instructions.

The concentrations of the immune soluble factors were determined using the Bio-Plex-200 system (Bio-Rad Corp., Hercules, CA, USA) and analysed with Bio-Plex Manager software (v.6; Bio-Rad). Results were reported as concentration (pg/mL).

### Statistical analysis

Contingency tables were used to summarize demographic and anthropometric data, oral hygiene habits, dental visit information, and clinical and inflammatory parameters.

Categorical variables were expressed as percentages (%), while continuous variables were assessed for normality using the Shapiro–Wilk test and reported as mean ± standard deviation (sd) or median and interquartile range (IQR), as appropriate. Cytokines and chemokines that were not normally distributed were log-transformed.

Differences between HC and all T1D subjects, as well as between T1D participants stratified by GCC and PGC, were evaluated using the χ^2^ test or Fisher’s test for categorical variables, and the t test or non-parametric Mann–Whitney U test for continuous variables. The same statistical approach was applied to compare clinical outcomes and salivary cytokine levels in T1D subjects with and without oral diseases.

Causal mediation analyses were conducted to assess whether the associations between salivary cytokine levels and oral pathologies (caries, gingivitis) were mediated by glycaemic control (defined by HbA1c values) or diabetes status. For mediation analysis, two linear regression models were specified: one modelling the mediator as a function of the exposure, and one modelling the outcome as a function of both the exposure and the mediator. Mediation effects were estimated using the mediation R package, with non-parametric bootstrapping (1.000 simulations) to compute 95% confidence intervals (CI). The Average Causal Mediation Effect (ACME), Average Direct Effect (ADE), Total Effect, and Proportion Mediated were reported.

Statistical significance was defined as p-value ≤ 0.05. All statistical analysis were performed using R software v.2023.12 (www.r-project.org).

## Results

### Subjects characteristics

In this study, 86 HC subjects and 72 individuals with T1D were enrolled. Among the T1D participants, 26 (36%) had GGC, while 46 (64%) had PGC. Age was significantly higher in the T1D group compared with HC (15.2 *vs.* 13.2 years, p-value = 0.002), especially in the PGC subgroup (15.6 years, PGC *vs*. HC p-value = 0.002). Moreover, T1D subjects showed a significantly higher BMI-SDS compared with HC (-0.1 *vs.* -0.4, p-value = 0.034). Stratification of T1D subjects by glycaemic control showed that PGC individuals had notably higher BMI-SDS values than HC (0.2, p-value = 0.013) (Table 1). No significant differences were found between GGC and PGC groups (data not shown).

**Table 1:** Demographic characteristics, oral hygiene practices, and oral assessment in type 1 diabetes (T1D) subjects, stratified by good glycaemic control (GGC) and poor glycaemic control (PGC), and healthy controls (HC

	HC (n = 86)	T1D (n = 72)	p-value T1D *vs.* HC	T1D GGC (n = 26)	p-value GGC *vs.* HC	T1D PGC (n = 46)	p-value PGC *vs.* HC	p-value GGC *vs.* PGC
**Demographic Characteristics**								
Sex, female %	53%	53%	1	54%	1	52%	1	1
Age years, mean ± sd	13.2 ± 4.5	15.2 ± 3.8	**0.002**	14.7 ± 3.9	0.11	15.6 ± 3.8	**0.002**	0.34
BMI-SDS, mean ± sd	-0.4 ± 1.1	-0.1 ± 1.1	**0.034**	-0.3 ± 0.8	0.60	0.2 ± 1.2	**0.013**	0.06
**Oral Hygiene Practices**								
Daily toothbrushing, yes %	100%	90%	**0.004**	100%	1	85%	**0.001**	0.09
Toothbrushing frequency, yes %								
1/day	2%	0%	0.64	0%	0.74	0%	0.67	0.30
2/day	19%	19%		11.5%		24%		
3/day	79%	81%		88.5%		76%		
Tools, yes %								
Manual toothbrush	75%	71%	0.59	58%	0.09	78%	0.83	0.10
Electric toothbrush	37%	37.5%	1	54%	0.17	28%	0.44	0.06
Mouthwash	10.5%	18%	0.25	15%	0.50	20%	0.19	0.76
Floss	23.5%	21%	0.71	15%	0.43	24%	1	0.55
Annual dental visit, yes %	95%	93%	0.73	96%	1	91%	0.45	0.45
Reason for visiting, yes %								
Check-up	79%	72%	0.34	72%	0.58	71%	0.38	1
Caries	54%	42%	0.14	44%	0.49	40%	0.18	0.80
Professional hygiene	47%	39%	0.40	36%	0.37	40%	0.57	0.80
Trauma	5%	6%	1	4%	1	7%	0.69	1
Pain	7%	6%	1	12%	0.44	2%	0.42	0.14
**Oral Assessment**								
pH saliva, mean ± sd	6.9 ± 0.3	6.8 ± 0.4	**0.001**	6.8 ± 0.2	0.07	6.7 ± 0.4	**0.003**	0.15
PI %, median (IQR)	38.8 (41.6)	46.1 (35.0)	0.21	43.8 (27.9)	0.54	50.5 (35.6)	0.19	0.48
BoP %, median (IQR)	6.9 (14.7)	10.7 (5.6)	**0.013**	10.4 (8.5)	0.71	11.3 (9.8)	**0.015**	0.07
Caries, yes %	28.5%	67%	** < 0.001**	54%	**0.033**	74%	** < 0.001**	0.14
Gingivitis, yes %	42%	76%	** < 0.001**	65%	**0.050**	83%	** < 0.001**	0.17
Caries + Gingivitis yes %	17%	56%	** < 0.001**	65%	**0.014**	91%	** < 0.001**	0.07

Data are shown as percentage (%), mean and standard deviation (mean ± sd) or median and interquartile range (IQR)

GGC = Good Glycaemic Control; PGC = Poor Glycaemic Control; BMI-SDS = BMI standard deviation scores; PI = Plaque Index; BoP = Bleeding on Probing

Differences among T1D and HC subjects were computed by$\chi$^2^ test or Fisher’s exact test for categorical variables, and by t test or Mann–Whitney U test for continuous variables

Significant p-values are shown in bold. Statistical significance was set at a p-value ≤ 0.05

### Oral hygiene and oral health in T1D and HC subjects

When comparing oral hygiene and annual dental care, 90% of T1D subjects reported brushing their teeth daily, compared with 100% of HC (p-value = 0.004). However, this difference was statistically significant only between PGC group and HC (p-value = 0.001), while no significant difference was observed between GGC and HC.

No significant differences were also observed in the frequency of daily tooth brushing, the methods used for oral hygiene, the frequency of annual dental visits or in the reasons for visits.

With regard to oral health, both groups presented neutral salivary pH, although the mean pH was significantly lower (i.e., more acid) in T1D subjects compared with HC (6.8 *vs.* 6.9, p-value = 0.001). In addition, T1D participants showed a higher percentage of BoP compared with HC (10.7% *vs.* 6.9%, p-value = 0.013), and a significantly higher prevalence of caries (67% *vs.* 28.5%, p-value < 0.001), gingivitis (76% *vs.* 42%, p-value < 0.001) and the presence of both conditions simultaneously (56% *vs.* 17%, p-value < 0.001). No differences were found in PI.

When comparing T1D subjects with GGC and PGC to HC and each other, PGC subjects exhibited a significantly lower salivary pH compared with HC (6.7 *vs.* 6.9, p-value = 0.003). GGC subjects also showed a lower pH (6.8) compared with HC, though this difference was not statistically significant. BoP values were elevated in both T1D groups (10.4% in GGC and 11.3% in PGC) compared with HC, but statistical significance was reached only in the comparison between PGC subjects and HC (p-value = 0.015). PI did not differ significantly among GGC and PGC.

The presence of oral diseases (caries, gingivitis, both caries and gingivitis) remained significantly higher in both T1D groups compared with HC (p-value ≤ 0.05). Notably, T1D subjects with PGC exhibited higher frequencies of caries (74%), gingivitis (83%), and co-occurrence of both conditions (91%) compared with GGC subjects (caries 54%, gingivitis 65%, both 65%). However, none of the comparison between GGC and PGC subgroups reached statistical significance. These results are reported in Table 1.

### Clinical characteristics of T1D subjects with or without oral health diseases

The clinical characteristics of T1D subjects were evaluated according to the presence of caries, gingivitis, or both oral diseases (Table 2).

**Table 2:** Association between oral diseases and clinical outcomes in type 1 diabetes (T1D) subjects

	No-caries (n = 24)	Caries (n = 48)	p-value	No-Gingivitis (n = 17)	Gingivitis (n = 55)	p-value	No-Caries + No-Gingivitis (n = 9)	Caries + Gingivitis (n = 40)	p-value
Age at onset years, mean ± sd	6.4 ± 3.7	8.7 ± 4.0	**0.018**	6.5 ± 3.7	8.4 ± 4.0	0.08	4.6 ± 1.9	8.7 ± 4.0	** < 0.001**
Disease duration years, median (IQR)	6.4 (8.4)	7.6 (6.4)	0.55	9.2 (6.3)	6.4 (7.0)	0.22	6.8 (8.0)	6.8 (6.4)	0.73
DKA at onset, yes %	25%	34%	0.62	31%	31%	1	22%	32%	0.70
BMI-SDS, mean ± sd	-0.1 ± 1.1	0.1 ± 1.1	0.43	-0.2 ± 1.1	0.1 ± 1.1	0.30	-0.5 ± 0.8	0.1 ± 1.1	0.07
HbA1c %, mean ± sd	7.1 ± 0.6	7.8 ± 1.3	**0.002**	7.2 ± 0.8	7.6 ± 1.2	0.13	6.9 ± 0.6	7.8 ± 1.3	**0.004**
HbA1c mmol/mol, mean ± sd	54 ± 6.3	61 ± 13.8	**0.002**	56 ± 9.0	60 ± 13.1	0.13	53 ± 6.1	62 ± 14.3	**0.004**
IDAA1c, median (IQR)	10.2 (1.1)	10.6 (1.7)	0.19	10.3 (1.1)	10.6 (1.7)	0.60	10.1 (0.6)	10.5 (1.9)	0.18
**Insulin Therapy**									
MDI	16.5%	35.5%	0.23	18%	33%	0.49	11%	37.5%	0.35
CSII	46%	31%		41%	34%		44.5%	30%	
HCL	37.5%	33.5%		41%	33%		44.5%	32.5%	
TC (mg/dL), mean ± sd	163 ± 26.0	169 ± 24.4	0.37	168 ± 28.8	166 ± 23.9	0.83	160 ± 14.6	167 ± 20.8	0.26
LDL-C (mg/dL), mean ± sd	91.1 ± 21.8	93.4 ± 20.8	0.68	91.0 ± 23.7	93.1 ± 20.3	0.75	88.2 ± 13.2	93.2 ± 18.1	0.36
HDL-C (mg/dL), mean ± sd	59.0 ± 12.0	60.9 ± 15.2	0.58	62.3 ± 17.6	58.8 ± 12.7	0.19	61.1 ± 9.14	56.1 ± 12.5	0.60
TG (mg/dL), median (IQR)	55.0 (22.5)	69.0 (36.0)	**0.046**	58.0 (19.0)	68.0 (27.5)	0.13	58.0 (12.0)	69.0 (29.3)	**0.027**
Blood Creatinine (mg/dL), mean ± sd	0.7 ± 0.2	0.7 ± 0.2	0.32	0.7 ± 0.2	0.7 ± 0.2	0.77	0.7 ± 0.3	0.7 ± 0.2	0.73
Urine Creatinine (mg/dL), median (IQR)	110 (102)	133 (118)	0.055	97.0 (89.8)	143 (111)	0.13	78.0 (51.5)	130 (115)	**0.013**
eGFR (mL/min/1.73m2), mean ± sd	131 ± 24.6	124 ± 25.9	0.27	127 ± 30.1	126 ± 24.5	0.90	135 ± 30.8	125 ± 25.8	0.42
ACR (mg/g), median (IQR)	4.8 (3.7)	5.9 (3.9)	0.43	6.1 (5.0)	5.4 (4.4)	0.33	6.1 (4.8)	5.9 (4.0)	0.98

Data are shown as percentage (%), mean and standard deviation (mean ± sd) or median and interquartile range (IQR)

DKA = Ketoacidosis; BMI-SDS = BMI standard deviation scores; IDAA1c = Lower Insulin-Dose Adjusted A1c; MDI = Multiple Daily Injections; CSII = Continuous subcutaneous insulin infusion; HCL = Hybrid Closed Loop; TC = Total Cholesterol; LDL-C = LDL Cholesterol; HDL-C = HDL Cholesterol; TG = Triglycerides; eGFR = estimated glomerular filtration rate; ACR = albumin-to-creatinine ratio

Differences among T1D subjects with and without caries, with and without gingivitis, or with and without both oral diseases were computed by $\chi$^2^ test or Fisher’s exact test for categorical variables, and by t test or Mann–Whitney U test for continuous variables

Significant p-values ​​are shown in bold. Statistical significance was set at a p-value ≤ 0.05

T1D subjects with caries had a significantly higher mean age at diabetes onset compared with those without caries (8.7 *vs.* 6.4 years, p-value = 0.018).

Additionally, subjects with caries showed higher mean of HbA1c levels (7.8% (62 mmol/mol) *vs.* 7.1% (54 mmol/mol), p-value = 0.002), higher triglycerides concentrations (69.0 *vs.* 55.0 mg/dL, p-value = 0.046), and elevated urinary creatinine levels (133 *vs.* 110 mg/dL), although the latter was only close to statistical significance (p-value = 0.055).

No statistically significant difference was found between T1D subjects with and without gingivitis. However, a trend toward higher median HbA1c (7.6% (60 mmol/mol) *vs.* 7.2% (55 mmol/mol)), triglycerides levels (68.0 *vs.* 58.0 mg/dL) and urinary creatinine (143 *vs.* 97.0 mg/dL) was showed in subjects with gingivitis.

When comparing T1D individuals with both caries and gingivitis to those without oral diseases, HbA1c levels (7.8% (62 mmol/mol) *vs.* 6.9% (52 mmol/mol), p-value = 0.004) and triglyceride levels (69.0 *vs.* 58.0 mg/dL, p-value = 0.027) were significantly higher in those with both oral conditions. Moreover, a significant difference was observed in urinary creatinine, with level averaging 130 mg/dL in subjects with both conditions compared with 78.0 mg/dL in those without (p-value = 0.013).

### Oral cytokines in T1D subjects with or without oral health diseases

The study also investigated salivary inflammatory profiles in T1D subjects according to the presence of oral diseases (Supplementary Table 1).

**Fig. 1:**
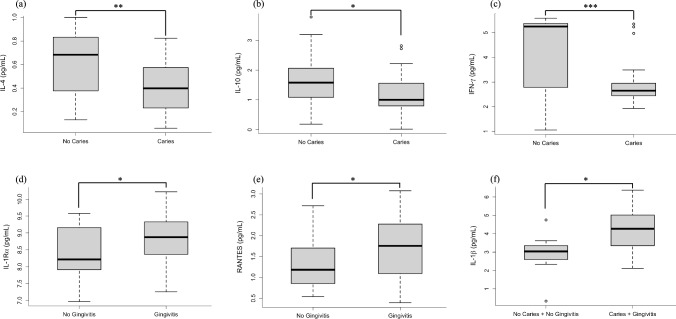
Association between salivary cytokine levels and oral diseases in type 1 diabetes (T1D) subjects. Salivary levels of interleukin (IL)-4 (**a**), IL-10 (**b**), and interferon-gamma (IFN-γ) (**c**) in T1D subjects with and without dental caries; IL-1 receptor antagonist (IL-1Ra) (**d**) and RANTES (**e**) in T1D subjects with and without gingivitis; IL-1β (**f**) in T1D subjects with and without both oral diseases (caries and gingivitis). All comparisons shown are statistically significant (* p-value < 0.05, ** p-value < 0.01, *** p-value < 0.001)

T1D subjects with caries exhibited significantly lower levels of IL-4 (0.5 *vs.* 0.9 pg/mL, p-value = 0.002), IL-10 (1.2 *vs.* 1.6 pg/mL, p-value = 0.022), and IFN-γ (2.8 *vs.* 4.4 pg/mL, p-value < 0.001) compared with T1D subjects without caries (Fig. 1a, b and c).

In contrast, gingivitis was associated with significantly increased levels of IL-1Ra (8.8 *vs.* 8.4 pg/mL, p-value = 0.045) and RANTES (1.8 *vs.* 1.3 pg/mL, p-value = 0.020) (Fig. 1d and e).

Finally, T1D subjects presenting both oral conditions showed significantly higher levels of IL-1β compared with those without oral diseases (4.1 *vs.* 2.9 pg/mL, p-value = 0.015) (Fig. 1f).

### Mediating role of glycaemic control and T1D

To explore the potential mediating role of glycaemic control or diabetes status in the association between salivary cytokines and oral health outcomes, causal mediation analyses were performed. When HbA1c was tested as a mediator, no mediation effect (ACME) for any cytokine-oral disease association emerged, suggesting that HbA1c did not mediate the relationships between IL-4, IL-10, IFN-γ, IL-1Ra, IL-1β, or RANTES and the presence of caries and/or gingivitis (data not shown).

Then, when diabetes status was tested as a mediator, a mediation effect was observed only for the association between IL-4 and caries, where diabetes significantly mediated the effect. In this case, both the ACME (estimate = -0.11, 95% CI: -0.21 to -0.03, p-value < 0.001) and the ADE (estimate = -0.27, 95% CI: -0.44 to -0.05, p-value = 0.008) were significant, with the proportion of the association between IL-4 and caries mediated by diabetes of approximately 29% (95% CI: 0.10–0.72, p-value < 0.001).

## Discussion

### Oral health in T1D subjects

This study highlights notable differences in oral health between children and young adults with T1D and HC, which cannot be attributed to disparities in oral hygiene habits.

Findings revealed a slightly more acidic salivary pH in T1D participants that in HC, although both groups maintained values within the neutral range. Even a moderate shift toward acidity may favour enamel demineralization and bacterial growth, thereby promoting caries development [31]. This is consistent with the observation of significantly higher rates of dental caries in T1D individuals, in agreement with previous studies [4, 32].

However, prior research has linked the increased caries prevalence in T1D individuals specifically to inadequate glycaemic control, with significant differences only observed between PGC and HC groups [33, 34]. Similarly, in this study, the presence of caries was associated with higher HbA1c levels, and caries were approximately 20% more prevalent in PGC compared with GGC subjects. Nonetheless, T1D groups had significantly higher caries prevalence than HC. These partially divergent results may be explained by the stricter HbA1c thresholds adopted in the present study to define GGC, compared with the aforementioned investigations, which considered HbA1c < 7.5% (58 mmol/mol) or < 8.0% (64 mmol/mol) as indicative of adequate control [33, 34].

This study also found that T1D subjects with caries had higher triglycerides levels compared with those without caries. This finding was confirmed by a study conducted by Subramaniam P. et al. (2015) [35], who observed a similar association in salivary triglycerides and suggested that increased lipid concentration in dental plaque might enhance the activity of glucosyl transferase, thereby increasing the cariogenic potential of oral microorganisms.

In addition, BoP, a marker of gingival inflammation, was significantly higher among T1D subjects, along with a greater prevalence of gingivitis, despite a similar oral hygiene degree (measured with the PI) in both groups. This result can suggest a predisposition in T1D subjects to develop inflammation. In fact, previous studies have described the inflammatory burden that diabetes imposes on the oral cavity, linking T1D in childhood with an increased risk of periodontal disease, considered one of the most frequent complications of T1D [6, 36].

Similar to caries, gingivitis has previously been associated with PGC. Indeed, hyperglycaemia can impair neutrophil function and collagen metabolism, hindering tissue repair, while elevated glucose levels in the gingival crevicular fluid promote microbial dysbiosis and periodontal damage [17, 34, 37].

Although a higher prevalence of gingivitis was observed in PGC subjects compared with GGC, this difference did not reach statistical significance, and no significant association emerged between HbA1c levels and the presence of gingivitis. Moreover, both T1D groups exhibited a significantly higher prevalence of gingivitis compared with HC.

In line with these findings, previous studies have also reported poorer gingival health and higher incidence of gingivitis in T1D subjects, regardless of glycaemic control [38, 39]. As noted earlier, discrepancies among studies may arise from differences in inclusion criteria or methodological approaches, including variations in HbA1c thresholds and the tools employed for oral health assessment.

Interestingly, T1D subjects presenting both caries and gingivitis exhibited poorer clinical outcomes, including suboptimal glycaemic control, elevated triglyceride levels, and a notable increase in urinary creatinine. Previous adult studies have reported link between periodontal disease or gingival bleeding and renal impairment [40, 41]. To our knowledge, this is the first study to document an association between oral diseases and renal alterations in children and young adults with T1D. Although markers of renal damage (ACR and eGFR) remained within normal ranges, the elevated urinary creatinine levels may serve as an early warning signal. These findings underscore the importance of integrated medical and dental care in this population and highlight the need for longitudinal studies to further explore relationship between oral health and diabetes-related complications in youth.

### Salivary cytokines profile in relation to oral disease in T1D

In this study, salivary cytokines were analysed to better understand the local inflammatory profile associated with oral diseases in individuals with T1D.

Notably, T1D subjects with caries showed significantly lower salivary concentrations of both anti-inflammatory cytokines, including IL-4, IL-10, as well as proinflammatory cytokines, such as IFN-γ, compared with T1D subjects without caries. These findings diverge from previous studies, which predominantly reported elevated levels of proinflammatory mediators in children with dental caries [42, 43]. The concomitant reduction of both anti- and proinflammatory cytokines in T1D cohort may indicate a dysregulated or exhausted immune profile specific to T1D. Indeed, T1D is known to involve a downregulation of the Th2 immune response, resulting in reduced production of cytokines, as IL-4 and IL-13 [44]. In line with this evidence, in the present study, mediation analysis revealed that T1D accounted for approximately one-third of the variance in the association between IL-4 and caries.

Findings of this study also reported that T1D subjects with gingivitis showed significantly higher salivary levels of RANTES. This chemokine is involved in the recruitment of inflammatory cells via interaction with its receptor and progenitor cells for RANTES have been identified in inflamed gingival tissue [45, 46], highlighting its role in the pathogenesis of periodontitis. This proinflammatory condition is in line with existing literature associating increased levels of proinflammatory interleukins with gingival inflammation [47].

Interestingly, these individuals also showed increased levels of IL-1Ra, an endogenous antagonist of IL-1β. Although IL-1Ra is thought to mitigate inflammation, its precise role in gingival homeostasis remains uncertain. Sawada et al. (2013) [47] similarly noted increased IL-1Ra expression in inflamed human gingival fibroblasts, though it is unclear how IL-1Ra may act in this context, nor whether it may contribute to reducing gingival inflammation.

Interestingly, in this work, T1D subjects affected by both caries and gingivitis showed markedly elevated levels of IL-1β, reinforcing its role in oral inflammation and periodontal disease among individuals with T1D [48].

Altogether, the cytokine profile observed in T1D individuals with oral disease appears to be distinct from that of HC with similar conditions (Supplementary Table 2). The cytokine profile also differed among healthy controls according to oral health status, with participants without oral diseases exhibiting a distinctive pattern of protective cytokines (i.e., IL-13, Monocyte Chemoattractant Protein-1) clearly different from that observed in T1D individuals.

Notably, this altered pattern, characterized by the simultaneous downregulation or upregulation of specific cytokines, is not consistently explained by either glycaemic control or diabetes status, as suggested by mediation analyses.

This supports the existence of a unique inflammatory signature in T1D, likely reflecting complex, diabetes-related immune dysregulation at the oral level. Consistent with this concept, evidence from immunotherapy research indicates that strategies aimed at modulating the autoimmune response against β-cells, including cytokine-directed therapies, may help preserve β-cell function and improve glycaemic control [49]. Together, these observations suggest that immune dysregulation in T1D is systemic and may also influence inflammatory responses at peripheral and mucosal sites, including the oral cavity, providing a conceptual link to the altered salivary cytokine profiles observed in our study.

### Limitations

This study has some limitations. For example, information on dietary habits and salivary flow was not available, although both factors may influence the development of oral diseases, particularly in subjects with T1D. Another limitation includes the use of self-reported hygiene behaviour, which may be subject to bias. Moreover, continuous glucose monitoring (CGM) data were not available, preventing the evaluation of short-term glycaemic fluctuations in relation to oral outcomes.

## Conclusions

The present study suggest that children and adolescents with T1D may be at increased risk of impaired oral health, as reflected by a higher prevalence of dental caries and gingivitis. These oral health deficits appear to be associated with adverse clinical outcomes, including suboptimal glycaemic control, elevated triglyceride levels, and increased urinary creatinine concentrations. Moreover, the study reveals a distinct salivary cytokine profile in T1D subjects with oral diseases, indicative of a dysregulated immune response involving both pro- and anti-inflammatory pathways.

These findings indicate that oral health may not only reflect local oral conditions but could also serve as an informative marker of broader metabolic and inflammatory complications.

Overall, these results highlight the potential importance of integrating systematic oral health assessment into routine diabetes management, independently of glycaemic control, to help prevent and mitigate broader metabolic and inflammatory complications. Future studies should explore whether targeted interventions to improve oral health can contribute to better glycaemic and metabolic outcomes in this population.

## Supplementary Information

Below is the link to the electronic supplementary material.

Supplementary file1 (DOCX 30 KB)

## Data Availability

The datasets generated during and/or analysed during the current study are available from the corresponding author on reasonable request.
