# Supplementary material for: Oral health and salivary inflammatory markers in children and adolescents with type 1 diabetes: A cross-sectional study
Source: J Endocrinol Invest. 2026 Jan 8;49(6):1403–12. doi: 10.1007/s40618-026-02808-4 (PMC13219055; doi:10.1007/s40618-026-02808-4)
Supplement: Supplementary file 1 — Supplementary file1 (DOCX 30 KB) [file 40618_2026_2808_MOESM1_ESM.docx]

**Oral health and salivary inflammatory markers in children and adolescents with type 1 diabetes: a case-control study**

Journal of Endocrinological Investigation

Eulalia Catamo, Gianluca Tornese, Chiara Navarra, Luana Aldegheri, Nunzia Zanotta, Manola Comar, Milena Cadenaro, Antonietta Robino.

**Corresponding authors:**

Gianluca Tornese, Institute for Maternal and Child Health – IRCCS Burlo Garofolo, via dell’Istria 65/1, 34137, Trieste, Italy, +390403785470, gianluca.tornese@burlo.trieste.it.

**Supplementary Table 1:** Association between salivary cytokines and oral diseases in type 1 diabetes (T1D) subjects, stratified by the presence of caries, gingivitis, or both.

| Cytokines | No-Caries  (n=24) | Caries  (n=48) | p-value | No-Gingivitis  (n=17) | Gingivitis  (n=55) | p-value | No-Caries +  No-Gingivitis  (n=9) | Caries+  Gingivitis  (n=40) | p-value |
| --- | --- | --- | --- | --- | --- | --- | --- | --- | --- |
| IL-1β,  mean±sd | 3.5±1.4 | 4.1±1.1 | 0.056 | 3.4±1.3 | 4.1±1.2 | 0.076 | 2.9±1.2 | 4.1±1.1 | **0.015** |
| IL-1Ra,  mean±sd | 8.9±0.6 | 8.7±0.8 | 0.20 | 8.4±0.8 | 8.8±0.7 | **0.045** | 8.5±0.6 | 8.8±0.8 | 0.35 |
| IL-2,  mean±sd | 1.7±0.6 | 1.5±0.6 | 0.14 | 1.5±0.6 | 1.6±0.6 | 0.61 | 1.6±0.5 | 1.5±0.5 | 0.71 |
| IL-4,  mean±sd | 0.9±0.5 | 0.5±0.3 | **0.002** | 0.6±0.4 | 0.7±0.4 | 0.23 | 0.6±0.4 | 0.5±0.3 | 0.57 |
| IL-6,  mean±sd | 2.2±1.0 | 2.3±1.1 | 0.63 | 1.9±1.1 | 2.4±1.1 | 0.11 | 1.9±1.1 | 2.4±1.1 | 0.26 |
| IL-8,  mean±sd | 6.7±0.8 | 6.3±1.0 | 0.15 | 6.2±1.0 | 6.5±0.9 | 0.33 | 6.5±1.0 | 6.4±1.0 | 0.85 |
| IL-10,  mean±sd | 1.6±0.8 | 1.2±0.7 | **0.022** | 1.2±0.6 | 1.4±0.8 | 0.39 | 1.4±0.5 | 1.2±0.7 | 0.41 |
| IL-13,  mean±sd | 0.5±0.3 | 0.4±0.2 | 0.099 | 0.4±0.2 | 0.4±0.3 | 0.58 | 0.4±0.2 | 0.4±0.2 | 0.72 |
| IL-17,  mean±sd | 1.9±0.6 | 1.6±0.7 | 0.053 | 1.6±0.6 | 1.7±0.7 | 0.52 | 1.6±0.6 | 1.6±0.7 | 1 |
| IFN-γ,  mean±sd | 4.4±1.4 | 2.8±0.7 | **<0.001** | 3.1±1.3 | 3.4±1.2 | 0.49 | 3.7±1.6 | 2.9±0.8 | 0.18 |
| TNF-α,  mean±sd | 3.7±1.3 | 3.4±1.1 | 0.29 | 3.1±1.1 | 3.6±1.2 | 0.11 | 3.1±0.9 | 3.4±1.1 | 0.28 |
| IP-10,  mean±sd | 5.2±1.9 | 4.9±1.7 | 0.49 | 5.2±1.8 | 4.9±1.8 | 0.60 | 5.3±2.2 | 4.8±1.8 | 0.59 |
| MCP-1,  mean±sd | 4.6±1.3 | 4.8±1.0 | 0.53 | 4.2±1.4 | 4.9±0.9 | 0.078 | 4.4±1.7 | 5.0±0.9 | 0.37 |
| MIP-1α,  mean±sd | 0.6±0.5 | 0.7±0.7 | 0.24 | 0.7±0.6 | 0.7±0.6 | 0.79 | 0.7±0.6 | 0.7±0.7 | 0.88 |
| RANTES,  mean±sd | 1.9±0.9 | 1.6±0.6 | 0.11 | 1.3±0.6 | 1.8±0.7 | **0.020** | 1.3±0.6 | 1.6±0.5 | 0.17 |

Data are shown as mean and standard deviation (mean±sd). All cytokines were expressed in pg/mL.

IL = Interleukin; IFN-γ =Interferon-γ; TNF-α = Tumor Necrosis Factor- α; IP-10 = Interferon-γ-inducible Protein-10; MCP-1 = Monocyte Chemoattractant Protein-1; MIP-1α = Macrophage Inflammatory Protein-1α

Differences among T1D subjects with and without caries, with and without gingivitis, or with and without both oral diseases were computed by t test.

Significant p-values ​​are shown in bold. Statistical significance was set at a p-value ≤ 0.05

**Supplementary Table 2:** Association between salivary cytokines and oral diseases in healthy controls (HC) subjects, stratified by the presence of caries, gingivitis, or both.

| Cytokines | No-Caries  (n=60) | Caries  (n=24) | p-value | No-Gingivitis  (n=50) | Gingivitis  (n=36) | p-value | No-Caries +  No-Gingivitis  (n=39) | Caries+  Gingivitis  (n=15) | p-value |
| --- | --- | --- | --- | --- | --- | --- | --- | --- | --- |
| IL-1β,  mean±sd | 3.2±1.0 | 3.3±1.0 | 0.67 | 3.1±1.0 | 3.5±1.0 | 0.053 | 3.1±1.2 | 4.1±1.0 | 0.22 |
| IL-1Ra,  mean±sd | 8.7±0.6 | 8.5±0.9 | 0.35 | 8.6±0.8 | 8.6±0.6 | 0.79 | 8.7±0.6 | 8.4±0.6 | 0.22 |
| IL-2,  mean±sd | 1.6±0.5 | 1.4±0.6 | 0.23 | 1.6±0.5 | 1.4±0.5 | **0.027** | 1.6±0.5 | 1.3±0.6 | 0.053 |
| IL-4,  mean±sd | 0.9±0.4 | 0.9±0.5 | 0.78 | 0.9±0.4 | 0.9±0.4 | 0.49 | 0.9±0.4 | 0.8±0.4 | 0.31 |
| IL-6,  mean±sd | 2.6±1.3 | 2.8±1.3 | 0.41 | 2.5±1.1 | 2.9±1.2 | 0.13 | 2.5±1.1 | 3.0±1.1 | 0.12 |
| IL-8,  mean±sd | 6.8±0.8 | 6.6±0.8 | 0.42 | 6.8±0.8 | 6.7±0.8 | 0.62 | 6.9±0.8 | 6.7±0.8 | 0.55 |
| IL-10,  mean±sd | 1.5±0.6 | 1.6±0.7 | 0.29 | 1.5±0.6 | 1.5±0.6 | 0.83 | 1.5±0.6 | 1.6±0.6 | 0.45 |
| IL-13,  mean±sd | 0.5±0.2 | 0.4±0.3 | 0.52 | 0.5±0.3 | 0.4±0.2 | 0.22 | 0.5±0.2 | 0.3±0.2 | **0.016** |
| IL-17,  mean±sd | 2.0±0.4 | 2.0±0.6 | 0.95 | 2.0±0.5 | 2.0±0.4 | 0.51 | 2.0±0.4 | 1.9±0.5 | 0.65 |
| IFN-γ,  mean±sd | 5.3±0.3 | 5.2±0.4 | 0.24 | 5.3±0.3 | 5.2±0.4 | 0.16 | 5.3±0.3 | 5.1±0.5 | 0.14 |
| TNF-α,  mean±sd | 3.6±0.9 | 3.5±0.9 | 0.84 | 3.6±0.9 | 3.6±0.9 | 0.88 | 3.5±0.9 | 3.4±0.8 | 0.75 |
| IP-10,  mean±sd | 4.5±1.6 | 4.3±1.8 | 0.59 | 4.5±1.7 | 4.5±1.7 | 0.93 | 4.6±1.7 | 4.7±1.8 | 0.91 |
| MCP-1,  mean±sd | 4.9±0.9 | 4.5±0.9 | 0.051 | 4.9±1.0 | 4.6±0.7 | 0.25 | 5.0±0.9 | 4.5±0.5 | **0.021** |
| MIP-1α,  mean±sd | 0.8±0.6 | 0.7±0.4 | 0.96 | 0.8±0.6 | 0.7±0.5 | 0.73 | 0.8±0.7 | 0.7±0.5 | 0.89 |
| RANTES,  mean±sd | 1.8±0.9 | 1.8±0.9 | 0.91 | 1.8±0.8 | 1.7±0.9 | 0.93 | 1.7±0.9 | 1.6±01.1 | 0.79 |

Data are shown as mean and standard deviation (mean±sd). All cytokines were expressed in pg/mL.

IL = Interleukin; IFN-γ =Interferon-γ; TNF-α = Tumor Necrosis Factor- α; IP-10 = Interferon-γ-inducible Protein-10; MCP-1 = Monocyte Chemoattractant Protein-1; MIP-1α = Macrophage Inflammatory Protein-1α

Differences among HC subjects with and without caries, with and without gingivitis, or with and without both oral diseases were computed by t test.

Significant p-values ​​are shown in bold. Statistical significance was set at a p-value ≤ 0.05
